# Transcriptomic and Metabolomic Analysis of the Response of Quinoa Seedlings to Low Temperatures

**DOI:** 10.3390/biom12070977

**Published:** 2022-07-12

**Authors:** Heng Xie, Qianchao Wang, Ping Zhang, Xuesong Zhang, Tingzhi Huang, Yirui Guo, Junna Liu, Li Li, Hanxue Li, Peng Qin

**Affiliations:** College of Agronomy and Biotechnology, Yunnan Agricultural University, Kunming 650201, China; 2020210159@stu.ynau.edu.cn (H.X.); 2020110028@stu.ynau.edu.cn (Q.W.); 2021110031@stu.ynau.edu.cn (P.Z.); 2020240160@stu.ynau.edu.cn (X.Z.); 2020240162@stu.ynau.edu.cn (T.H.); 2020240137@stu.ynau.edu.cn (Y.G.); 2021110026@stu.ynau.edu.cn (J.L.); 2019210130@stu.ynau.edu.cn (L.L.); 2021210172@stu.ynau.edu.cn (H.L.)

**Keywords:** Quinoa, metabolome, transcriptome, low-temperature stress, response mechanism

## Abstract

Quinoa, a cool-weather high-altitude crop, is susceptible to low-temperature stress throughout its reproductive phase. Herein, we performed broadly targeted metabolic profiling of quinoa seedlings to explore the metabolites’ dynamics in response to low-temperature stress and transcriptome analysis to determine the underlying genetic mechanisms. Two variants, namely, Dian Quinoa 2324 and Dian Quinoa 281, were exposed to temperatures of −2, 5, and 22 °C. A total of 794 metabolites were detected; 52,845 genes, including 6628 novel genes, were annotated using UPLC-MS/MS analysis and the Illumina HiSeq system. Combined with morphological indicators to resolve the mechanism underlying quinoa seedling response to low-temperature stress, the molecular mechanisms of quinoa changed considerably based on temperature exposure. Soluble sugars heavily accumulated in plants with cold damage and changes in regulatory networks under freeze damage, such as the upregulation of α-linolenic acid metabolism and a reduction in energy substrates, may explain the spatial patterns of biosynthesis and accumulation of these metabolites. Genes that are actively expressed during cold responses, as revealed by co-expression analyses, may be involved in the regulation thereof. These results provide insights into the metabolic factors in quinoa under low-temperature stress and provide a reference for the screening of quinoa varieties resistant to low temperature.

## 1. Introduction

The transcriptome is an important research tool used to characterize an entire set of transcripts and obtain gene expression profiles of various organisms [1], whereas the metabolome is the embodiment of an organism’s phenotype; Fiehn has previously used metabolomics in 2002 as a qualitative and quantitative research method to study metabolites [2]. The physiological and biochemical responses to adversity in plants are complex; hence, it is difficult to elucidate the exact mechanisms underlying plant responses by only studying the transcriptome or metabolome alone. The correlation analysis of the intrinsic links between genes and metabolites is an important approach to studying plant stress resistance [3,4,5].

Abiotic stress adversely affects normal crop growth, thereby causing significant economic losses. A low temperature is one of the most common abiotic stresses; freezing damage is frequently seen in plants from various countries, such as Australia, Korea, Canada, the United States, India, and Peru [6]. For instance, in China, spring frost is severe in low-altitude areas, such as the Yangtze River basin and the Yunnan-Guizhou plateau. Cold stress generally leads to a decline in plant nutrient uptake, reduced photosynthesis, destruction of the formative layer, and toxicity due to hydrogen peroxide accumulation in the plant body [7]. Low-temperature stress can alter anther metabolic pathways and induce sterile pollen production [8] and can lead to sterile ovules, flower failure, fertilization collapse, and low seed quality, resulting in lower plant yield [9]. Further, cold and freezing stress can cause plant wilting, dwarfing, and fading green [10], among other adverse effects.

Quinoa (*Chenopodium quinoa* Willd.), an annual dicotyledonous herb in the subfamily Chenopodioideae of the family Amaranthaceae, is a cool-weather, high-altitude crop native to the Andes [11]. Quinoa seeds are gluten-free, low in glucose, and have substantial levels of essential amino acids, fiber, and lipids, making it a whole grain, which is referred to as “cereal gold” by the Food and Agriculture Organization [12,13]. Quinoa is one of the few crops that can tolerate some degree of freezing stress based on various factors, such as plant variety, freezing duration, soil microenvironment, environmental relative humidity, and fertility stage at the time of freezing [14]. Freezing damage in quinoa at the seedling stage affects the nutrient uptake and growth of quinoa seedlings, prolongs the seedling period, and causes dwarfing of the nutritive tissues, affecting the final yield. The cold resistance of quinoa varieties of different colors has been studied from physiological and biochemical points of view [15]. The adaptability of quinoa varieties to low temperatures varies considerably [16]. It is now widely believed that the main survival mechanisms of quinoa under freezing stress are its presence of supercooled liquid that avoids freezing and the high concentration of soluble sugars in quinoa that reduces coagulation and mean lethal temperature [17]. Studies on changes in physiological mechanisms of quinoa under low-temperature stress have not been reported; however, understanding the mechanisms of quinoa response to low-temperature stress is important for breeding low-temperature tolerant varieties and widening the area under quinoa cultivation.

Therefore, in this study, we sequenced and analyzed the transcriptomes and metabolomes of the low-temperature-sensitive quinoa line (Dian Quinoa 2324) and a low-temperature-tolerant quinoa line (Dian Quinoa 281) exposed to freezing (−2 °C) until semi-lethality, cold (5 °C), or room temperature (22 °C). Our study aimed to explore the key metabolic pathways and regulatory factors and elucidate the possible molecular mechanisms underlying quinoa responses to low-temperature stress. This study provides new insights into the mechanism related to quinoa response to low-temperature stress, and the results can provide a theoretical basis for molecular breeding of quinoa and standardized production of high-yielding and high-quality quinoa under low temperature, and broaden the direction of selection and development of low-temperature tolerant quinoa varieties.

## 2. Materials and Methods

### 2.1. Experimental Materials and Treatments

Thirty quinoa high-generation lines, selected independently by Yunnan Agricultural University, were used as the primary screening material. They were planted at the modern agricultural education and research base of the University in Xundian County, Kunming (102°41′ E, 25°20′ N). Uniform seeds were sown in three 50-hole seedling trays (54 × 28 × 12 cm), in three replicates. In the first stage, the seeds were managed according to conventional cultivation techniques (average temperature, 22 °C; sunshine duration, approximately 10 h; sowing depth, 2–3 cm; loamy soil: humus: organic fertilizer = 1:1:1). When the seedlings attained the six-leaf stage, the two treatment groups were transferred to a greenhouse at −2 °C and 5 °C, respectively, with other conditions being the same as those for field planting. Most of the terminal leaves were observed to freeze with the −2 °C treatment, which was considered the best time to sample (11 h). Based on plant growth and the degree of leaf freeze death, the most cold-tolerant (Dian Quinoa 281) and least cold-tolerant (Dian Quinoa 2324) of these lines were selected as samples. The leaves and roots were sampled, rapidly frozen in liquid nitrogen, and stored at −80 °C. The leaf samples were used for metabolome and transcriptome sequencing and quantitative reverse-transcription polymerase chain reaction (RT-qPCR) analysis. Three biological replicates and three technical replicates were included in this study. Here, Y1 represents Dian Quinoa 2324 and Y2 represents Dian Quinoa 281; where the treatment with A indicates −2 °C treatment (freezing damage), labeled as AY group; B represents 5 °C treatment (cold damage), labeled as BY group; C represents 22 °C treatment (room temperature), labeled as CY group.

### 2.2. Morphological Data Collection

Dian Quinoa 2324 and Dian Quinoa 281 were examined separately after 11 h of treatment (three replicates) to determine the plant height, leaf area, dry and fresh weight, root to crown ratio, root length, and leaf color of quinoa seedlings (leaf color is indicated by ‘L’ indicates brightness; ‘a’ indicates red and green values; negative numbers indicate green values; the smaller the green brightness). Plant height (distance from the base to the tip of the topmost spreading leaf) and leaf area were measured using Vernier calipers and the TPYX-A (Zhejiang, China, https://www.tpyn.net (accessed on 10 July 2021)) crop leaf morphometer, respectively. The GXY-A (Zhejiang, China, https://www.tpyn.net (accessed on 12 July 2021)) root analysis system was used for the root system measurements. Leaf color was scanned with a color reader (CR-20). Seedlings were killed at 110 °C for 30 min and dried to a constant weight at 85 °C to determine the dry and fresh weights, and the root-to-crown ratio.

### 2.3. Metabolite Extraction Detection and Qualitative and Quantitative Analyses

Sample preparation, protein extraction, broadly targeted metabolite identification, and qualitative quantification were performed at Wuhan Metware Biotechnology (Wuhan, China) (http://www.metware.cn/ (accessed on 6 June 2021)). Freeze-dried samples were analyzed using a liquid chromatography–electrospray ionization–tandem mass spectrometry (MS) system (CNWBOND Carbon-GCB SPE Cartridge, 250 mg, 3 mL; Shanghai Ampere Scientific Instruments) based on an in-house Metware database. Metabolite quantification was conducted by multiple-reaction monitoring. A multivariate statistical analysis was used to maximize the retention of raw information. The data were simplified and downscaled to create numerical models for inter-group analyses and to determine associated differences using the built-in statistical prcomp function [18] of the R software (www.r-project.org/ (accessed on 2 July 2021)) [19]. Heat maps were drawn by the R software pheatmap, and the differential accumulation of metabolites (DAMs) between samples was analyzed by hierarchical clustering. OPLS-DA was used to extract the components in the independent variable X and the dependent variable Y, followed by the screening of differential variables [20]. The variable importance in projection (VIP) of the OPLS-DA model for multivariate analysis was obtained and combined with the P-value and fold change to further screen for differential metabolites [20]. Metabolites were considered significant if they differed by a factor of ≥2 or ≤0.5 in the control and treatment groups, respectively. The differential metabolites were further calibrated and annotated in the KEGG database (https://www.kegg.jp/kegg/compound/ (accessed on 22 June 2021)) [21]; their significance was determined using the P-value of the hypergeometric test.

### 2.4. Transcriptome Sequencing and Data Analysis

RNA extraction and detection, library construction, sequencing, and bioinformatics analysis were performed at Wuhan Metware Biotechnology, Ltd. (Wuhan, China, www.metware.cn (accessed on 6 June 2021)). Total RNAs were removed from Dian Quinoa 2324 and Dian Quinoa 281 using the Plant RNA Kit (Omega, Shanghai, China) according to the company’s directions. A Qubit^®^ RNA Assay Kit and Qubit^®^ 2.0 Fluorometer (Life Technologies, Foster City, CA, USA) and RNA Nano 6000 Assay Kit and Bioanalyzer 2100 system (Agilent Technologies, CA, USA) were used to measure RNA concentration and detect RNA integrity, respectively. Initial quantification was performed using a Qubit 2.0 Fluorometer, and the library quality was evaluated using an Agilent Bioanalyzer 2100 system. The clustering of the index-coded samples was performed on a cBot Cluster Generation System using. Clustering of the index-coded samples was performed on a cBot Cluster Generation System using fastp v 0.19.3 to filter the original data. Gene pairings were calculated using features v1.6.2, followed by FPKM for each gene based on gene lengths. TruSeq PE Cluster Kit v3-cBot-HS (Illumia) TruSeq PE Cluster Kit v3-cBot-HS (Illumia) differential expression between two groups was analyzed using DESeq2 v1.22.1. The *P*-values were corrected using the Benjamini–Hochberg method and a corrected *P*-value (false discovery rate, FDR) > 0.05 and |log2foldchange (FC)| ≥ 1 were used as thresholds for significant differential expression. Kyoto Encyclopedia of Genes and Genomes (KEGG) and gene ontology (GO) term enrichment analyses were based on a hypergeometric test; for KEGG, a hypergeometric distribution test was conducted with paths as units, whereas for GO, it was performed based on GO terms.

### 2.5. Combined Transcriptomic and Metabolomic Analysis

Differentially expressed metabolites (DAMs) were combined with differentially expressed genes (DEGs) to analyze the mechanisms underlying the quinoa responses to low-temperature stress. DAMs and DEGs were simultaneously mapped on a KEGG pathway map. A correlation analysis was performed on genes and metabolites detected in each sample group. Pearson correlation coefficients (PCCs) between genes and metabolites were calculated using the COR program in R. Network plots were used to represent the correlation coefficients of metabolites and genes with PCC > 0.8 in each group. A canonical correlation analysis (CCA) was used to reflect the overall correlation between the transcriptomes and metabolomes. An orthogonal two-partial least squares (O2PLS) model was established using all DEGs, while DAMs and variables with higher correlations and weights in the different datasets were selected based on loadings plots to filter out important variables affecting the other omics [22].

### 2.6. RT-qPCR Analysis

We used BeaconDesign v7.9 to design primer pairs specific for 15 selected genes (Supporting Information, Table S1). qPCRs were run in an ABI 7500 system (Applied Biosystems, Foster City, CA, USA) using PerfectStart^TM^ SYBR qPCR Supermix (TransGen Biotech, Beijing, China). The thermal cycles were as follows: 94 °C for 30 s, 40 cycles of 94 °C for 5 s, and 60 °C for 30 s. Relative gene expression levels were calculated by the 2^−ΔΔCt^ method, using the *TUB-6* gene as an internal reference [23].

## 3. Results

### 3.1. Changes in Agronomic Traits in Quinoa Seedlings under Low-Temperature Stress

**Figure 1:**
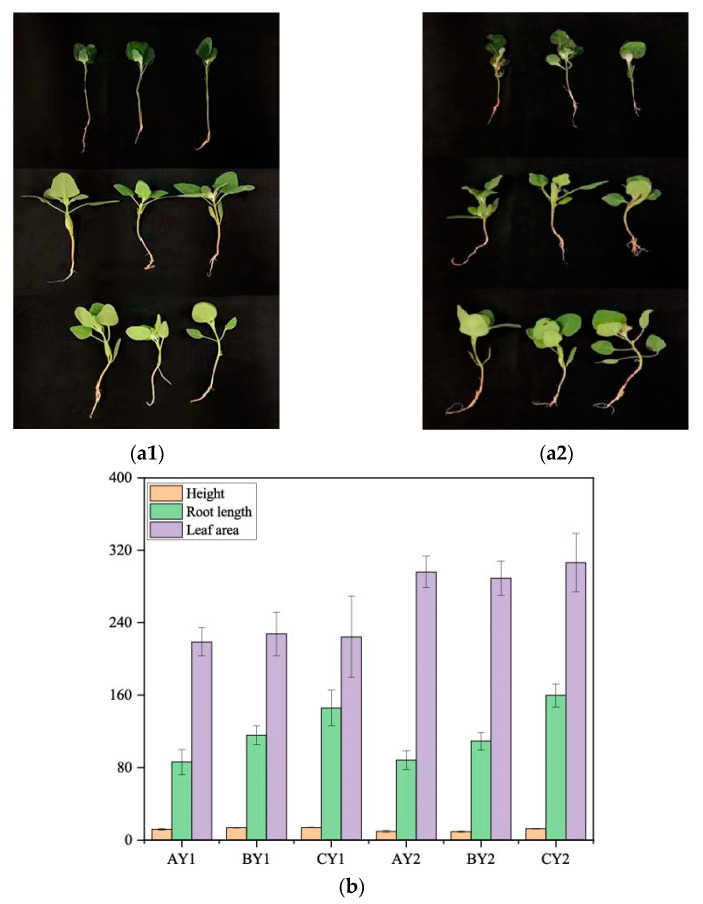
Phenotypic map of quinoa under different treatments: (**a1**) Dian Quinoa 2324 (**a2**) Dian Quinoa 281. from top to bottom, AY1, BY1, CY1 vs. AY2, BY2, CY2 treatments. (**b**) Height (cm), root length (mm), leaf area (mm^2^). Error bars indicate SEs.

Changes in morphological indicators of Dian Quinoa 2324 and Dian Quinoa 281 under 11 h of low-temperature stress were compared (Table 1, Figure 1a). The low-temperature-tolerant line 281 showed limited changes in plant height under freezing and cold stress and had a more stable relative water content and root-to-crown ratio under low temperature, especially under freezing, than line 2324 (Figure 1b).

### 3.2. Transcriptome Changes in Quinoa Seedlings under Low-Temperature Stress

We divided the plants into six groups, AY1, BY1, CY1, AY2, BY2, and CY2, with three biological replicates in each group. In total, 128.05 Gb of clean sequencing data were obtained, with 19–24 Gb per sample, a Q30 score > 91%, and FDR < 0.03% (ab.S1). The genome alignment ratio was >90%, indicating that the data were reliable for a subsequent analysis. We annotated 52,845 genes in total, including 6628 novel genes, 38,129 genes in the KEGG database, 39,959 genes in the GO database, 47,614 genes in the euKaryotic Orthologous Groups (KOG) database, and 38,938 genes in the Pfam database.

**Figure 2:**
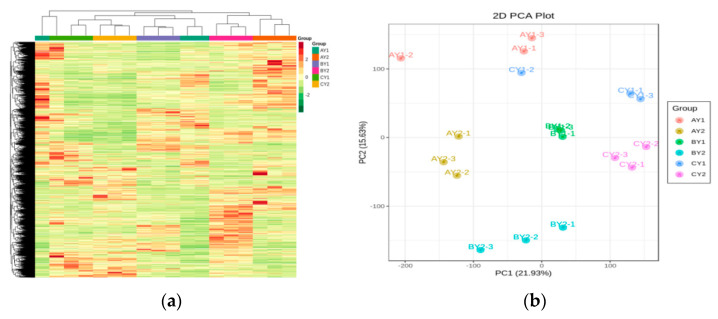

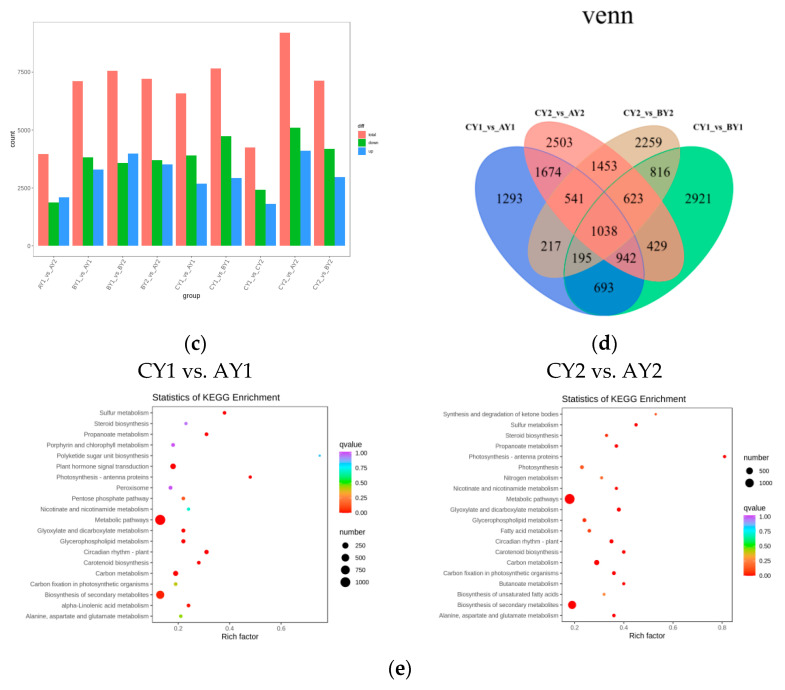
Analysis of transcriptomes differences in quinoa lines under low-temperature stress: (**a**) heat map of differential gene clustering; (**b**) PCA plot of genes; (**c**) statistical map of DEGs; (**d**) Wayne plot of differential genes for low-temperature treatment; (**e**) enrichment scatter plot.

In this part of the study, CY was defined as the control group and AY and BY as the freeze-treated and cold-treated groups, respectively. The centralized and normalized FPKM expressions of the differential genes were extracted to construct a hierarchical clustering heat map (Figure 2a). Subsequently, a principal component analysis (PCA, Figure 2b) was performed to show that differences between sample groups were greater than within-group differences and indicated good within-group biological reproducibility. There were some differences between low-temperature stress (−2 °C and 5 °C) and the normothermic condition, and the differences were greater at −2 °C, suggesting more severe damage at this temperature. We screened for DEGs between all the possible pairs of treatments based on |log2FC| ≥ 1 and FDR < 0.05. We thus found 6593 DEGs (2686 upregulated [up], 3907 downregulated [down]) in CY1 vs. AY1; 7657 DEGs (2931 up, 4726 down) in CY1 vs. BY1; 9203 DEGs (4099 up; 5104 down) in CY2 vs. AY2; 7142 DEGs (2963 up, 4179 down) in CY2 vs. BY2; 7103 DEGs (3288 up, 3815 down) in BY1 vs. AY1; 7217 DEGs (3512 up, 3705 down) in BY2 vs. AY2; 3957 DEGs (2095 up, 1862 down) in AY1 vs. AY2; 7554 DEGs (3979 up, 3575 down) in BY1 vs. BY2; 4242 DEGs (1813 up, 2429 down) in CY1 vs. CY2 (Figure 2c).

When we compared the DEGs of CY1 vs. AY1, CY2 vs. AY2, CY1 vs. BY1, and CY2 vs. BY2 (Figure 2d), we found that 1038 DEGs were expressed in both varieties under cold and freezing stress. Presumably, these genes are involved in the low-temperature stress response of quinoa and are closely related to its tolerance to low temperatures. In CY1 vs. AY1 and CY2 vs. AY2, only a small proportion of DEGs was common between cold and freezing stresses, with freezing stress (−2 °C) inducing a larger number of genes than cold stress (5 °C). To analyze the DEGs further, we subjected them to GO and KEGG enrichment analyses. Thirty-one pathways were significantly (*p* < 0.05) enriched in DEGs in both CY1 vs. AY1 and CY2 vs. AY2, mainly including sugar metabolism, propionate metabolism, photosynthesis, phytohormone biosynthesis, fatty acid biosynthesis, phospholipid metabolism, and amino acid biosynthesis. Among them, “Polyketide sugar unit biosynthesis” and “Aflatoxin biosynthesis” were only enriched in low temperature-sensitive lines (CY1 vs. AY1). “BSynthesis and degradation of ketone bodies” was only enriched in low-temperature tolerant lines (CY2 vs. AY2). The top 20 pathways that were the most significantly enriched in both comparisons are shown in Figure 2e. The GO analysis showed that the DEGs were mostly involved in biological processes, with significant enrichment in the secondary active transmembrane transporter protein activity, α-amino acid metabolism, chlorophyll metabolism, photosynthesis, chloroplast, plastid endosomes, pigment metabolism, and carboxylic acid catabolism (Supporting Information, Figure S1).

### 3.3. Metabolomic Changes in Quinoa Seedlings under Low-Temperature Stress

**Figure 3:**
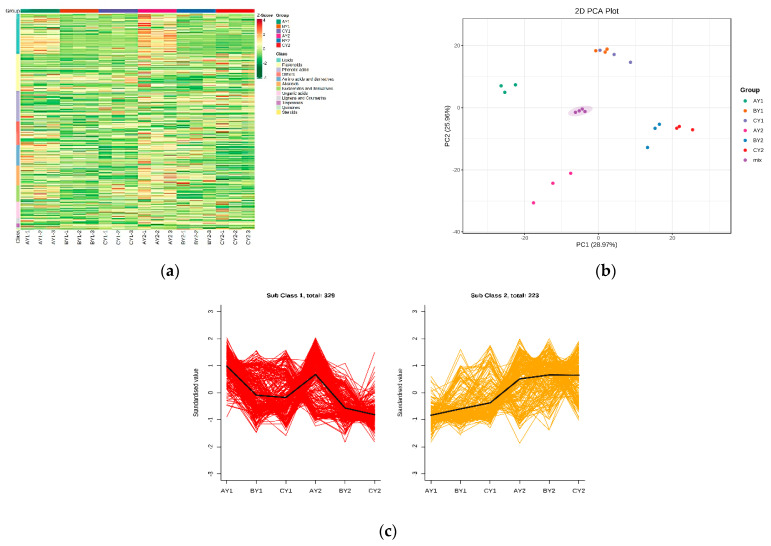
Analysis of metabolome differences in quinoa lines under low-temperature stress: (**a**) overall cluster plot of samples; (**b**) PCA plot of metabolites; (**c**) K-means plot of differential metabolites.

To gain a more comprehensive and in-depth understanding of the effects of low-temperature stress on quinoa seedlings, metabolomic analyses were carried out using various targeted metabolomics techniques. In total, 794 metabolites were detected, which were grouped into 12 categories, including 148 lipids, 137 flavonoids, 112 phenolic acids, 77 amino acids and their derivatives, 73 alkaloids, 60 nucleic acids and their derivatives, 59 organic acids, 21 lignans and coumarins, 15 terpenoids, 5 quinones, 1 steroid, and 86 others (Figure 3a). PCA showed significant interspecific differences between the freeze treatment (group A) and the control (group C) (Figure 3b) and suggested slight seedling damage at 5 °C and significant damage at −2 °C, which is consistent with the transcriptomic results.

We used an orthogonal partial least squares discriminant analysis (OPLS-DA) to mine differential metabolites, and the contribution of each metabolite in the OPLS-DA model was assessed by variable importance in the projection (VIP), using |log2FC| ≥ 1 and VIP ≥ 1 as thresholds. In CY1 vs. AY1, 234 significant DAMs were screened, 185 of which were upregulated and 49 downregulated. Six categories of DAMs were found to contain more than 10 DAMs: lipids (90, 38.4%), amino acids and their derivatives (38, 16.2%), alkaloids (26, 11.1%), nucleotides and their derivatives (19, 8.1%), organic acids (17, 7.2%), and phenolic acids (15, 6.4%). In CY2 vs. AY2, there were 264 significant DAMs, 233 of which were upregulated and 31 downregulated. Again, six categories contained more than 10 DAMs: lipids (98, 37.1%), amino acids and their derivatives (40, 15.1%), alkaloids (29, 10.9%), nucleotides and their derivatives (28, 10.6%), organic acids (19, 7.1%), and phenolic acids (16, 6%). In CY1 vs. BY1, 118 DAMs were screened, 60 of which were upregulated and 58 were downregulated. Five categories had more than 10 DAMs: amino acids and their derivatives (25, 21.1%), alkaloids (23, 19.4%), organic acids (18, 15.2%), and nucleotides and their derivatives (16, 13.5%). In CY2 vs. BY2, there were 122 significant DAMs, 88 of which were upregulated and 34 were downregulated. Six categories had more than 10 DAMs: amino acids and their derivatives (26, 21.3%), alkaloids (24, 19.6%), flavonoids (16, 13.1%), organic acids (13, 10.6%)), lipids (12, 9.6%), and nucleotides and their derivatives (10, 8.1%).

To compare the response to freezing (−2 °C) with that at room temperature (22 °C), we conducted differential metabolite co-expression analysis of CY1 vs. AY1 and CY2 vs. AY2. Among the top 10 upregulated metabolites, the top 9 significantly increased metabolites were lipids, a large proportion of which were free fatty acids, with the most significant change observed for lysophospholipids (Table 2). Lysophospholipid–signaling lipids that can be hydrolyzed from membrane-associated glycerophospholipids and released into the extracellular space where they can be recognized by extracellular receptors and initiate signaling pathways [24]. Under low-temperature treatment at 5 °C, the top 10 DAMs between both lines included 3 flavonoids, 2 organic acids, 1 nucleotide and its derivatives, 1 amino acid and its derivatives, 1 quinone lipid, and 1 other class.

Next, we compared the relative content trends of the metabolites in the different treatment groups. The relative contents of all DAMs identified in all treatment group comparisons according to the screening criteria were subjected to Z-score normalization followed by K-means clustering, which revealed two major trends for all DAMs (Figure 3c, Supporting Information, Table S2). We then subjected the DAMs identified to KEGG pathway enrichment analysis, using *p* < 0.05 as a threshold. The pathways significantly enriched in CY1 vs. AY1 and CY2 vs. AY2 co-expression were: aminoacyl tRNA biosynthesis; thioglucoside biosynthesis, valine, leucine, and isoleucine degradation, linoleic acid metabolism, amino acid biosynthesis, propionic acid metabolism, monobactam biosynthesis synthesis, and 2-oxocarbonyl acid metabolism. To clarify the overall trends in the KEGG metabolic pathways, we performed differential abundance analysis (Supporting Information, Figure S2) on the pathway maps of CY1 vs. AY1 and CY2 vs. AY2. The results revealed that both propionate metabolism and photosynthesis were significantly downregulated.

### 3.4. Joint Transcriptomic and Metabolomic Analyses of the Quinoa Low-Temperature-Stress Response Mechanisms

We integrated DEGs and DAMs into KEGG pathways and screened for pathways that were enriched in both DEGs and DAMs, using a threshold of *p* < 0.05. The pathways that were significantly enriched in CY1 vs. BY1 and CY2 vs. BY2 were ABC transporters, starch and sucrose metabolism, pyrimidine metabolism, phenylalanine, tyrosine, and tryptophan biosynthesis. A nine-quadrant plot of DEG and DAM correlations based on correlation coefficient values > 0.8 showed that most DEGs were consistent with the DAM patterns, with genes being upregulated and metabolites remaining unchanged or being downregulated, and that positive gene regulation was dominant over negative regulation in genes affecting metabolic changes (Supporting Information Figure S3).

Under low temperatures, plants accumulate a large amount of cellular soluble sugars to enhance their cold tolerance [25]; therefore, we next focused on trends in quinoa soluble sugars under low-temperature exposure using combined transcriptome and metabolome data. In CY1 vs. BY1 and CY2 vs. BY2, starch and sucrose pathways were significantly enriched at both the transcriptome and metabolome levels (*p* ≤ 0.01), and d-glucose-6-phosphate, d-fructose-6-phosphate, glycoside 5′-diphosphate-d-glucose, and the key intermediate glucose-1-phosphate were significantly increased in both treatments. The increase in d-glucose was not significant, and no significant changes in starch content were observed.

**Figure 4:**
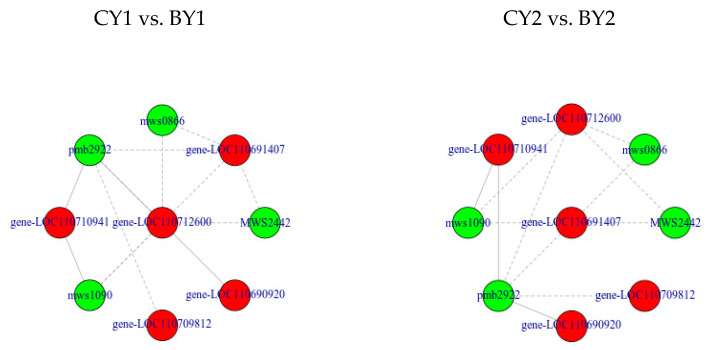
Correlations between genes and differential metabolites: Correlation network diagram, where metabolites are marked in green and genes in red, with solid lines representing positive correlations and dashed lines representing negative correlations.

To investigate the underlying mechanism, we correlated DEGs with significantly changed soluble sugars (Table 3). LOC110712600, LOC110691407, LOC110710941, LOC110709812, and LOC110690920 were highly correlated (|PCC| > 0.8) with soluble sugar content (Figure 4). We hypothesized that these five genes play important roles in regulating the soluble sugar synthesis pathway under cold stress, and we found that two genes, LOC110691407 and LOC110712600, encoding β-glucosidase [EC:3.2.1.21] and endoglucanase [EC:3.2.1.4], respectively, were positively correlated with increases in d-fructose-6-phosphate, d-glucose-6-phosphate, glucose-1-phosphate, and uridine 5′-diphosphate-d-glucose, and with the formation of l-proline (PCC = 0.825). We centralized and normalized the genes using FPKM and found that the |log2FC| values for LOC110691407 and LOC110712600 were greater in CY2 vs. AY2 (cold-tolerant line Dian 281) than in CYI vs. BY1, and these genes were more actively expressed in cold-tolerant line under low temperatures.

### 3.5. Potential Mechanism Underlying the Changes in Energy Substrates of Quinoa under Freezing Cold Stress

The following pathways were significantly enriched in both CY1 vs. AY1 and CY2 vs. AY2: propionic acid metabolism, α-linolenic acid metabolism, nicotinic acid, and nicotinamide pathways. These findings may elucidate the response mechanism of quinoa seedlings to severe freezing damage induced at −2 °C. The combined transcriptome metabolome results indicated that cold injury at −2 °C directly affects the energy supply and enzyme substrates of quinoa plants, which in this condition accumulate large amounts of unsaturated fatty acids that are toxic to the cells, causing the plants to senesce and die. In this study, we combined more significant metabolic pathways associated with both DEGs and DAMs after freeze treatment: propanoate metabolism, nicotinate and nicotinamide metabolism, and alanine, aspartate, and glutamate metabolism. The biosynthetic pathway mainly includes nicotinamide adenine dinucleotide (NAD+), amide adenine dinucleotide phosphate (NADP+), β-nicotinamide mononucleotide, nicotinamide, nicotinic acid, 6-hydroxynicotinic acid, nicotinic acid ribonucleotide, dihydroxyacetone phosphate, succinic acid, gamma-aminobutyric acid, and L-glutamic acid.

**Figure 5:**
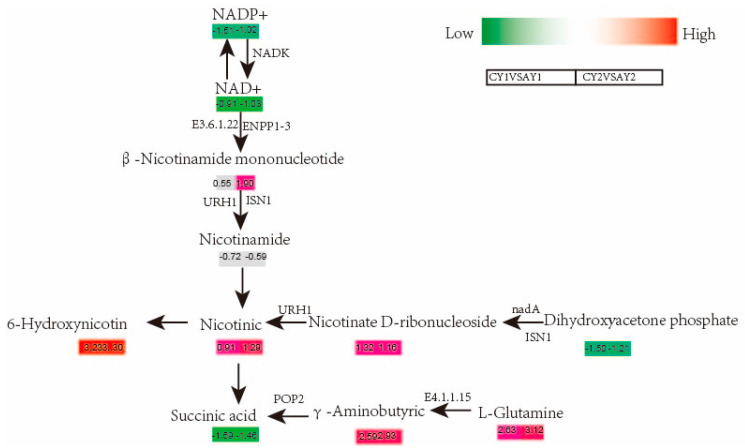
Key metabolism map of nicotinamide under freezing in quinoa: gene expression levels are expressed as FPKM values followed by calculation of their log2FC for different controls, metabolite levels are expressed as log2FC values for different controls, left indicates CY1VSAY1, right indicates CY2VSAY2. NADP+, nicotinamide adenine dinucleotide phosphate; NAD+, nicotinic acid adenine dinucleotide.

The mechanisms underlying the formation of these substances in relation to metabolic pathways have been mapped (Figure 5). NAD+ and its phosphorylated form, NADP+, are important energy converters and signaling molecules in all organisms, and in plants, these pyridine nucleotides have various functions, ranging from the central energy metabolism to key regulatory roles in development and immunity [26]. The key energy substrates NADP+, succinic acid, and dihydroxyacetone phosphate were all significantly downregulated upon freezing (AY), whereas the other nicotinamide derivatives significantly accumulated, and the |Log2FC| values were higher in CY1 vs. CY1 than in CY2 vs. AY2, indicating more stable changes in energy substrates in the low-temperature-tolerant line (Figure 5). Five genes, LOC110706353, LOC110687828, LOC110729149, LOC110705708, LOC110702874, and LOC110700051, were highly correlated with succinic acid, NADP+, and dihydroxyacetone phosphate (Table 4). Interestingly, LOC110729149 and LOC110700051, which are involved in plant growth and energy homeostasis, were highly correlated (|PCC| > 0.8) with large amounts of lysophosphatidyl, free amino acids, and glycosides.

### 3.6. Analysis of the Lipid Bilayer Phase Changes in Cell Membranes Based on the α-Linolenic Acid Metabolic Pathway

**Figure 6:**
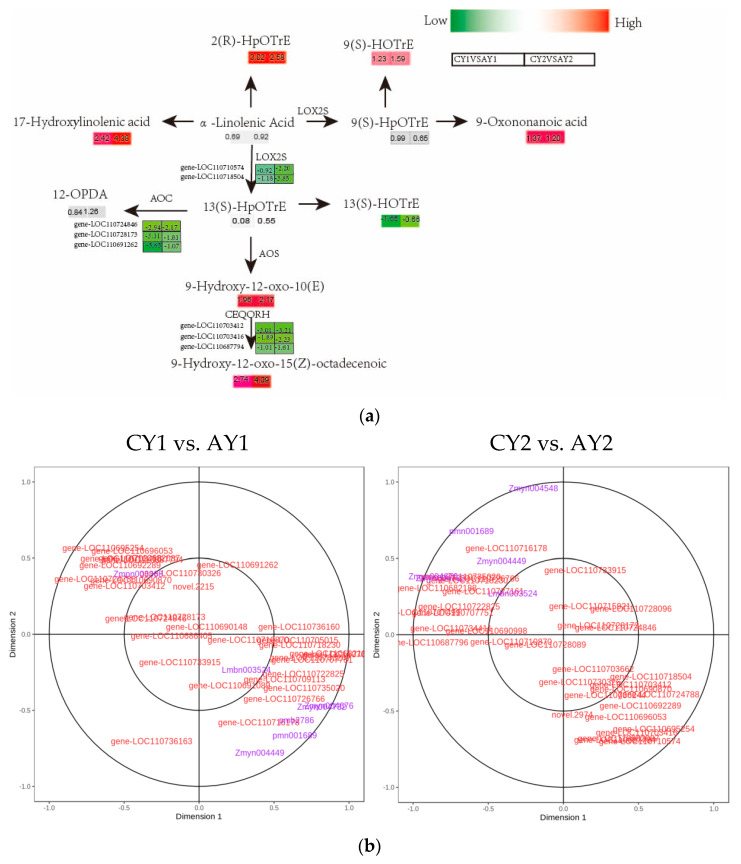
Comparative analysis of α-linolenic acid metabolism and related genes under freezing stress in quinoa. (**a**) Key metabolic pathways of α-linolenic acid during freezing in quinoa: gene expression levels are expressed as FPKM values followed by calculation of log2FC for different controls, and metabolite levels are expressed as log2FC values for different controls. LOX, lipoxygenase; LOS, hydroperoxide dehydratase; LOC, allene oxide cyclase; (**b**) metabolites of α-linolenic acid metabolism and the CCA of the gene, with metabolite index (purple) and gene ID (red).

It has been shown that direct damage to the cell membrane system, such as that caused by sub-zero temperatures, reduces membrane fluidity and causes changes in the membrane lipid bilayer phase, resulting in lipid degradation and release from the membrane [27,28]. As a common fatty acid, linolenic acid occupies a central position in lipid metabolism, especially in plant leaves, where its synthesis as a major membrane lipid component is prioritized. Linolenic acid, which belongs to the ω-3 family of polyunsaturated fatty acids, has two crystalline forms, α and γ, with α-linolenic acid being the dominant form [29]. We analyzed the mechanism underlying the changes in the α-linolenic acid metabolic pathway under freezing conditions (−2 °C) in quinoa by screening for enriched DEGs and DAMs. In total, 11 DAMs were enriched in CY1 vs. AY1, 7 of which were significant (log2FC > 1), with 32 associated DEGs. A total of 11 DAMs were enriched in CY2 vs. AY2, 6 of which were significant (log2FC > 1), with 34 associated DEGs. A metabolite-to-gene regulatory network was constructed based on the α-linolenic acid metabolic pathway in the KEGG pathway (Figure 6a). The 17-hydroxylinolenic acid, 9-oxonononanoic acid, and octadecenoic acid substantially increased in this metabolic reaction, and octadecenoic acid was found to be significantly elevated in both CY1 vs. AY1 and CY2 vs. AY2. In α-linolenic acid metabolism of quinoa, LOX regulates two metabolic nodes, LOC and LOS catalyze one metabolic node each, and α-linolenic acid is converted to fatty acid hydroperoxides.

Although the pathways controlling α-linolenic acid biosynthesis have been identified, it is not clear which genes play a key role in regulating the linoleic and unsaturated fatty acid biosynthetic pathways. Therefore, we performed CCA of the tissue contents of these compounds and gene expression in the relevant biosynthetic pathways to identify candidate genes. It was revealed that the 17-hydroxylinolenic acid and 9-hydroxy-12-oxo-15(Z)-octadecenoic acid contents were associated with LOC110692289, encoding secreted phospholipase A2 ([EC:3.1.1.4]), and LOC110682087, encoding 12-oxophosphodienoate reductase ([EC:1.3.1.42]), respectively; both transcripts were significantly correlated in the expression (Figure 6b, Supporting Information, Table S3).

### 3.7. RT-qPCR

Fifteen genes randomly selected from candidate DEGs involved in starch and sucrose metabolism, nicotinate and nicotinamide metabolism, and α-linolenic acid metabolism were analyzed by RT-qPCR to validate the RNA-sequencing-based transcriptome data. The RT-qPCR results for the candidate genes were broadly consistent with the relative transcript abundances found in the transcriptomic analysis (Supporting Information, Figure S4), validating that transcriptome data.

## 4. Discussion

Quinoa is an ancient crop of Andean origin that is known for its high nutritional value and excellent resistance to adversity. Commonly grown at high altitudes, quinoa is susceptible to low-temperature stress, and there is a wide low-temperature stress tolerance range among quinoa varieties [14]. Low-temperature stress can seriously affect normal crop development, and quinoa shows different levels of sensitivity to low temperatures at different stages of reproduction. Low-temperature stress at the seedling stage generally results in weak seedlings, delaying reproduction [30], and in severe cases, it causes extensive seedling mortality, ultimately affecting quality and yield.

In this study, we screened two lines, Dian Quinoa 281 and Dian Quinoa 2324, for DEGs/DAMs under low-temperature stress. DAMs and DEGs induced at 5 °C were found to be mainly involved in starch and sucrose metabolism, and those induced at −2 °C in nicotinate and nicotinamide metabolism and α-linolenic acid metabolism. The regulation of crop responses to low-temperature stress is complex and diverse and often involves multiple response pathways to stabilize the system in order to adapt to environmental stress. We found that the d-fructose-6-phosphate, d-glucose-6-phosphate, glucose-1-phosphate, and uridine 5′-diphosphate-d-glucose contents in starch and sucrose metabolism were significantly increased under cold stress. It is generally accepted that the soluble sugar content of plants can be increased after exposure to low temperatures to elevate the osmotic pressure in the cytosol and, thus, prevent dehydration and solidification of the protoplasm [31,32]. Under −2 °C treatment, we found significant decreases in the nicotinic acid and nicotinamide metabolic precursors NAD+ and NADP+. The NAD+ precursor vitamin B3, which is involved in various intra- and intercellular processes that regulate cell metabolism and stress and immune responses to physiological or pathological signals [33,34]. Gakière et al. reported that Arabidopsis nicotinamide and nicotinic acid pools are strongly associated with photosynthesis and respiration. NAD+ levels are reduced and senescence-related transcripts are upregulated. Altered NAD levels lead to premature senescence in plants, demonstrating the importance of balanced NAD+ metabolism for normal plant growth and development [35]. Dihydroxyacetone phosphate is an integral part of various sugar metabolisms entering the glycolytic process, and is an intermediate in the regeneration of ribulose 1,5-diphosphate from C6 and C3 sugars in the dark reaction of photosynthesis, It plays an important role in the energy response system [36,37]. The large amount of energy generated by succinate entering the tricarboxylic acid (TCA) cycle enhances plant resistance to abiotic stresses [38]. It was hypothesized that the decline in energy-related substrates, such as NAD+, NADP+, succinate, and dihydroxyacetone phosphate caused insufficient energy backfill in the system, dysregulating the plant metabolic network, in turn leading to abnormal crop growth and development.

Low subzero temperatures can cause direct damage to the cell membrane system, such as direct injury from low temperatures, reducing membrane fluidity and causing changes in the physical phase of membrane lipids [39]. We combined transcriptome and metabolome to show that quinoa activates the fatty acid oxidative metabolic pathway of α-linolenic acid metabolism under freezing stress. α-linolenic acid metabolic flux may represent a gateway to the biosynthesis of multiple unsaturated fatty acids [40], and the reaction products of downstream genes, such as LOX, AOS, and AOC can influence JA synthesis [41]. Regulation of α-linolenic acid to form JA under chilling stress affects the biosynthesis of unsaturated fatty acids and thus protects cells [42]. Notably, lysophosphatidylcholine 19:2 was significantly increased in quinoa seedlings exposed to 5 °C. The lipid composition of quinoa under the −2 °C treatment was significantly different from that under the 5 °C treatment. Lysophosphatidylcholine is hydrolyzed by lysophosphatidyl acyltransferase and increases at low temperatures, improving membrane fluidity [43]. We hypothesize that under freezing, lysophosphatidylcholine and lysophosphatidyl phosphatidylethanolamine increase rapidly, enhancing cell membrane fluidity, and the synthesis of polyunsaturated fatty acids through the alpha-linolenic acid metabolic pathway, causing changes in the physical phase of the cell membrane, improved quinoa tolerance to low temperatures.

It is important to understand the inter-regulation of metabolites and to recognize the genes that play key roles in metabolite synthesis, transport, and degradation. We speculate that the genes LOC110712600 and LOC110691407 play key roles in the synthesis of d-fructose-6-phosphate, d-glucose-6-phosphate, glucose-1-phosphate, and uridine 5′-diphosphate-d-glucose in quinoa under cold stress and that their active expression may render quinoa more resilient to cold stress. LOC110729149 and LOC110700051, which are involved in the energy reflux system, were also highly expressed, and we suspect that these two genes play important roles in regulating energy homeostasis. The enzymes encoded by LOC110682087, LOC110695254, and other genes are actively involved in the generation of α-linolenic acid from octadecenoic acid, and we suspect that these genes have important roles in the stabilization of the cell membrane structure. The response mechanisms of quinoa under low-temperature stress are complex and diverse, and further biochemical or biological studies on the functions of these candidate genes are required to better understand the interplay of pathways under low-temperature stress. However, in the absence of further information, it is difficult to confirm the functionality of candidate genes to identify candidate key genes for future studies. The interactions between several key pathways under low-temperature stress should also be investigated in the future.

## 5. Conclusions

In this study, we combined transcriptomics and metabolomics to investigate the response mechanisms of seedlings of two quinoa lines under low-temperature stress. We analyzed the expressions of differential metabolites in quinoa under cold and freezing damage, with lipids being the most significantly upregulated under freezing stress. Quinoa responds to low temperatures from a combination of aspects, maintaining its metabolism by accumulating soluble sugars under cold damage, and responding to stress by regulating the metabolism of energy substrates and α-linolenic acid in freezing damage. Combined DEG and DAM analysis showed that LOC110712600, LOC110691407, LOC110729149, LOC110700051, LOC110692289, LOC110682087, and LOC110695254 were closely related to the low-temperature stress response of quinoa. The metabolic and transcriptomic findings of this study provide important insights into the growth and development of quinoa under low-temperature stress, particularly in understanding the response mechanism under short and sudden frost damage. We believe that these findings will help in the screening of good quinoa varieties for low-temperature tolerance.

## Figures and Tables

**Table 1:** Agronomic traits of quinoa under different temperature treatments.

Sample Name	Height (cm)	Root Length (cm)	Leaf Area (mm^2^)	Relative Moisture Content	Root-to-Crown Ratio	Leaf Color Brightness (L)	Leaf Color Red-Green Value (a)
	M ± SD	M ± SD	M ± SD				
AY1	11.9 ± 1.31 ^a,b,c^	86.34 ± 23.63 ^c^	218.87 ± 26.22 ^a^	90.0%	2.78%	36.9	−6.8
BY1	13.7 ± 0.75 ^a,b^	115.68 ± 17.75 ^b,c^	227.60 ± 40.60 ^a^	91.1%	3.16%	35.5	−7.0
CY1	13.9 ± 0.46 ^a^	145.78 ± 33.21 ^a,b^	224.48 ± 76.14 ^a^	91.4%	3.30%	42.2	−7.6
AY2	9.7 ± 1.96 ^c^	88.51 ± 17.58 ^c^	295.91 ± 29.58 ^a^	91.4%	2.74%	40.1	−7.8
BY2	9.3 ± 1.30 ^b,c^	109.20 ± 16.30 ^b,c^	289.24 ± 32.26 ^a^	92.6%	2.43%	41.6	−7.4
CY2	12.6 ± 0.31 ^a,b,c^	159.74 ± 21.56 ^a^	306.44 ± 54.63 ^a^	92.3%	3.10%	48.7	−9.5

Note: M ± SD is mean ± standard deviation, different lowercase letters indicate significant differences at the level of 0.05 (*p* < 0.05).

**Table 2:** Top 10 DAMs in freezing vs. normothermia.

Class I	Class II	Compounds	Formula	Value
Lipids	LPC	LysoPC 19:2	C27H52NO7P	0.014
	LPE	LysoPE 18:0	C23H48NO7P	0.039
		LysoPE 16:3	C21H38NO7P	0.008
	Free fatty acids	9,12-Octadecadien-6-Ynoic Acid	C18H28O2	0.069
		9,10,13-Trihydroxy-11-Octadecenoic Acid	C18H34O5	0.015
		9,12,13-Trihydroxy-10,15-octadecadienoic acid	C18H32O5	0.019
		9,10,11-Trihydroxy-12-octadecenoic acid	C18H34O5	0.017
		13(S)-HODE;13(S)-Hydroxyoctadeca-9Z,11E-dienoic acid	C18H32O3	0.021
		9S-Hydroxy-10E,12Z-octadecadienoic acid	C18H32O3	0.023
Phenolic acids	Phenolic acids	Vanillin acetate	C10H10O4	0.019

Note: formula: molecular formula of the substance; compounds: English name of the substance; Class I: Class I category of the substance; Class II: Class II category of the substance; *p*-value: *p*-value of the significance test.

**Table 3:** Metabolite–gene correlations.

Compound	KEGG	Gene ID	PCC
D-Fructose-6-phosphate	K01188 beta-glucosidase [EC:3.2.1.21]	LOC110691407	−0.836
	K01179 endoglucanase [EC:3.2.1.4]	LOC110712600	−0.801
D-Glucose-6-phosphate	K01188 beta-glucosidase [EC:3.2.1.21]	LOC110691407	−0.82
	K01179 endoglucanase [EC:3.2.1.4]	LOC110712600	−0.802
Glucose-1-phosphate	K01177 beta-amylase [EC:3.2.1.2]	LOC110710941	0.801
	K01179 endoglucanase [EC:3.2.1.4]	LOC110712600	−0.809
	K01188 beta-glucosidase [EC:3.2.1.21]	LOC110691407	−0.841
Uridine 5′-diphosphate-D-glucose	K01179 endoglucanase [EC:3.2.1.4]	LOC110712600	−0.852
	K19891 glucan endo-1,3-beta-glucosidase 1/2/3 [EC:3.2.1.39]	LOC110690920	0.811
	K01179 endoglucanase [EC:3.2.1.4]	LOC110709812	−0.891
	K01177 beta-amylase [EC:3.2.1.2]	LOC110710941	0.856
	K01188 beta-glucosidase [EC:3.2.1.21]	LOC110691407	−0.904

**Table 4:** Key substance and gene correlation coefficients.

Compound	KEGG	Gene ID	PCC
Succinic acid	K01240 uridine nucleosidase [EC:3.2.2.3]	LOC110706353	−0.869
	K03517 quinolinate synthase [EC:2.5.1.72]	LOC110687828	0.912
	K03426 NAD+ diphosphatase [EC:3.6.1.22]	LOC110729149	0.842
NADP	K00858 NAD+ kinase [EC:2.7.1.23]	LOC110705708	0.804
		LOC110702874	0.832
	K00278 L-aspartate oxidase [EC:1.4.3.16]	LOC110700051	0.841
Dihydroxyacetone phosphate	K00278 L-aspartate oxidase [EC:1.4.3.16]	LOC110700051	0.921

## Data Availability

All relevant data can be found within the manuscript and its Supporting Materials. The datasets generated and analyzed during the current study are available from the corresponding author upon reasonable request.

## Supplementary Materials

The following supporting information can be downloaded at: https://www.mdpi.com/article/10.3390/biom12070977/s1.
