# Supplementary material for: Transcriptomic and Metabolomic Analysis of the Response of Quinoa Seedlings to Low Temperatures
Source: Biomolecules. 2022 Jul 12;12(7):977. doi: 10.3390/biom12070977 (PMC9312504; doi:10.3390/biom12070977)
Supplement: Supplementary file 1 [file biomolecules-12-00977-s001.zip › Fig.S1.pdf]

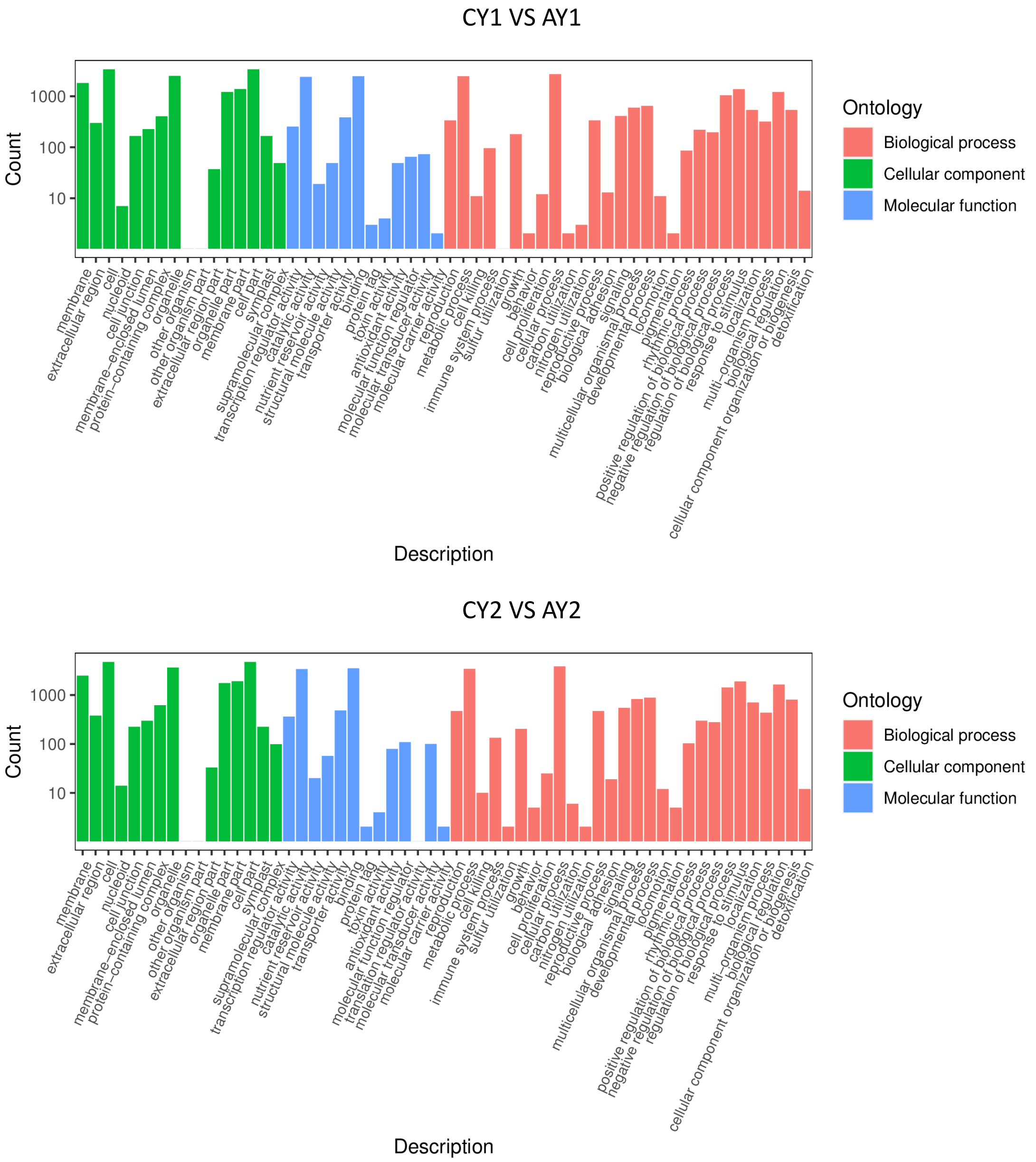

Figure S1: Classification chart of secondary entries of differential genes,The horizontal coordinate indicates the secondary GO entry and the vertical coordinate indicates the number of differential genes in the GO entry.
