# Supplementary material for: Transcriptomic and Metabolomic Analysis of the Response of Quinoa Seedlings to Low Temperatures
Source: Biomolecules. 2022 Jul 12;12(7):977. doi: 10.3390/biom12070977 (PMC9312504; doi:10.3390/biom12070977)
Supplement: Supplementary file 1 [file biomolecules-12-00977-s001.zip › Fig.S3.pdf]

CY1 VS BY1

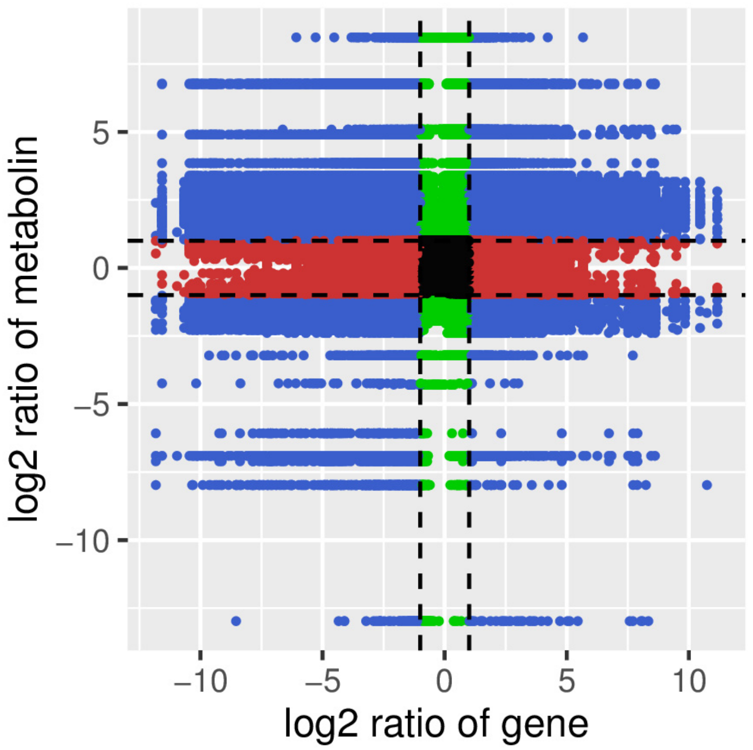

CY2 VS BY2

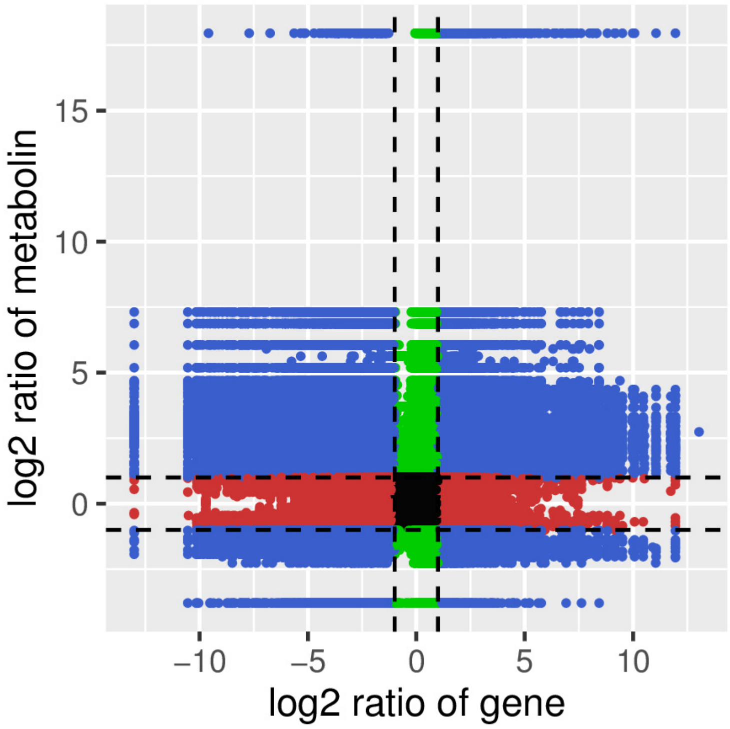


Figure S3: Correlation analysis nine-quadrant diagram: showing the number of fold differences in metabolites of genes with Pearson correlation coefficients greater than 0.8 in each difference subgroup.
