# Supplementary material for: Transcriptomic and Metabolomic Analysis of the Response of Quinoa Seedlings to Low Temperatures
Source: Biomolecules. 2022 Jul 12;12(7):977. doi: 10.3390/biom12070977 (PMC9312504; doi:10.3390/biom12070977)
Supplement: Supplementary file 1 [file biomolecules-12-00977-s001.zip › Fig.S4.pdf]

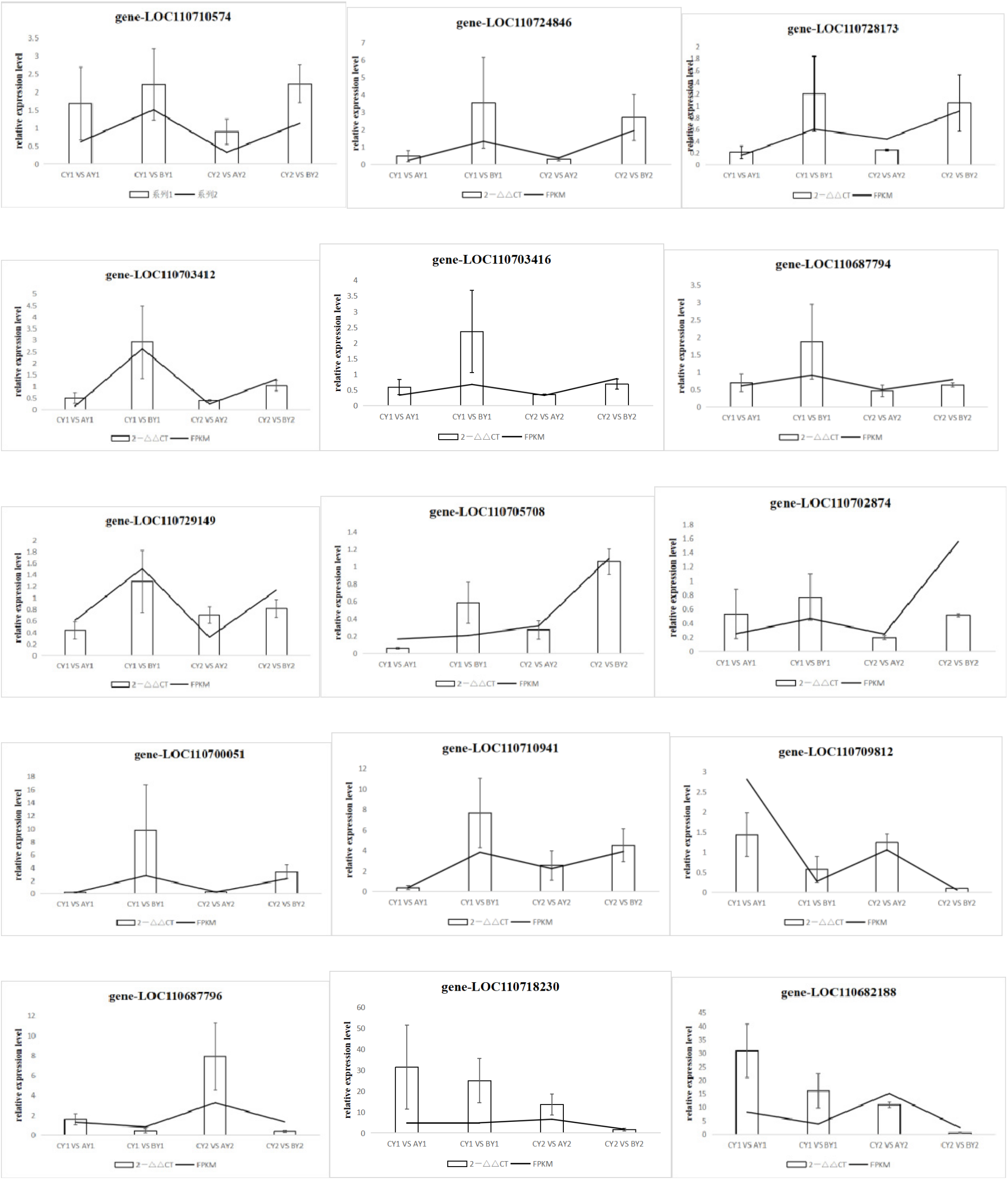

Figure S4: Line graphs indicate the transcript abundances measured by transcriptome sequencing based on FPKM values. Bar graphs represent the relative expression measured by RT-qPCR expressed in $2^{-\Delta\Delta Ct}$. Data are averages of three replicates and three biological replicates. Error bars indicate SEs.
