# Supplementary figures and images for: Transcriptomic and Metabolomic Analysis of the Response of Quinoa Seedlings to Low Temperatures
Source: Biomolecules. 2022 Jul 12;12(7):977. doi: 10.3390/biom12070977 (PMC9312504; doi:10.3390/biom12070977)

CY1 VS AY1

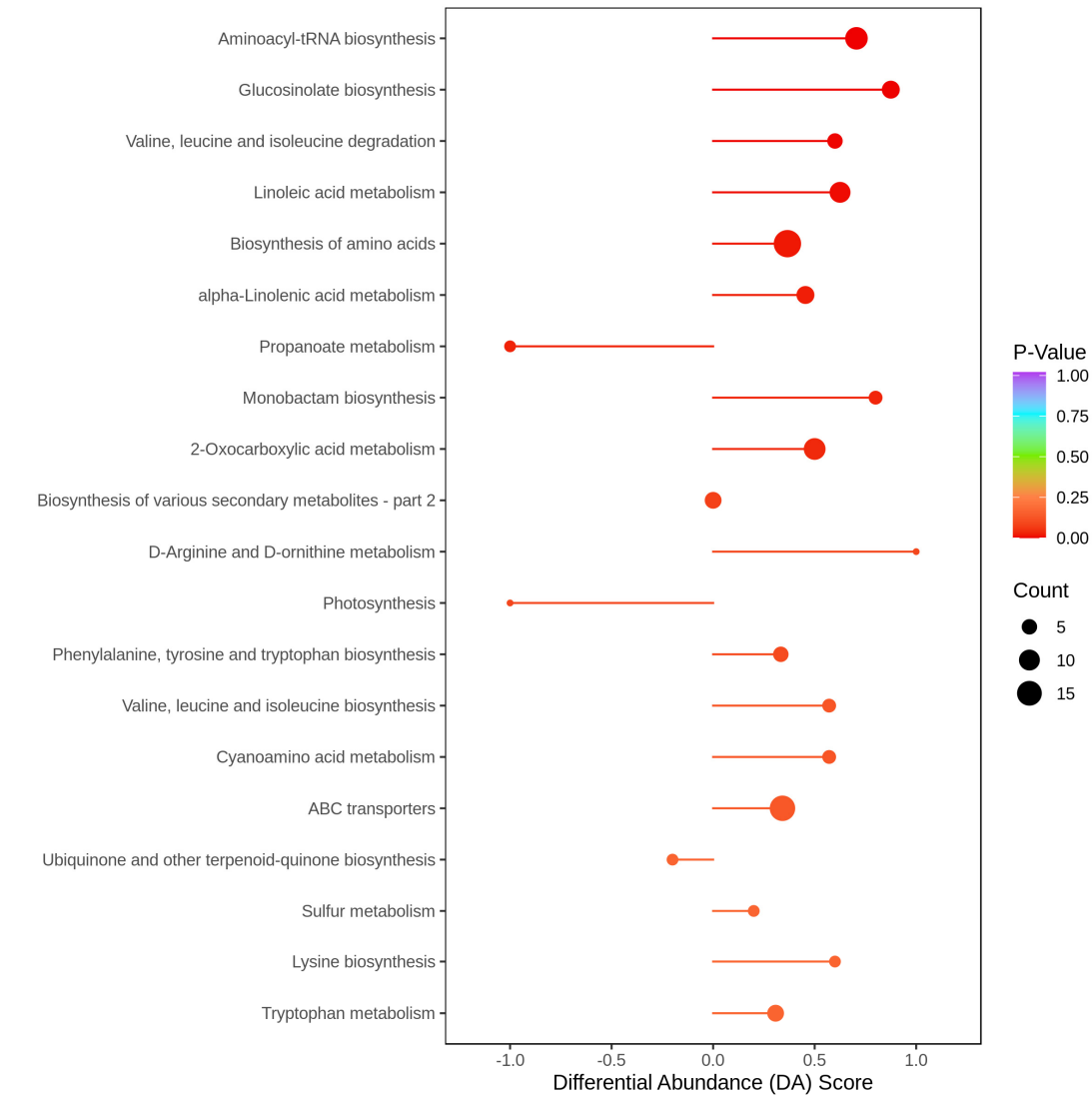

CY VS AY2

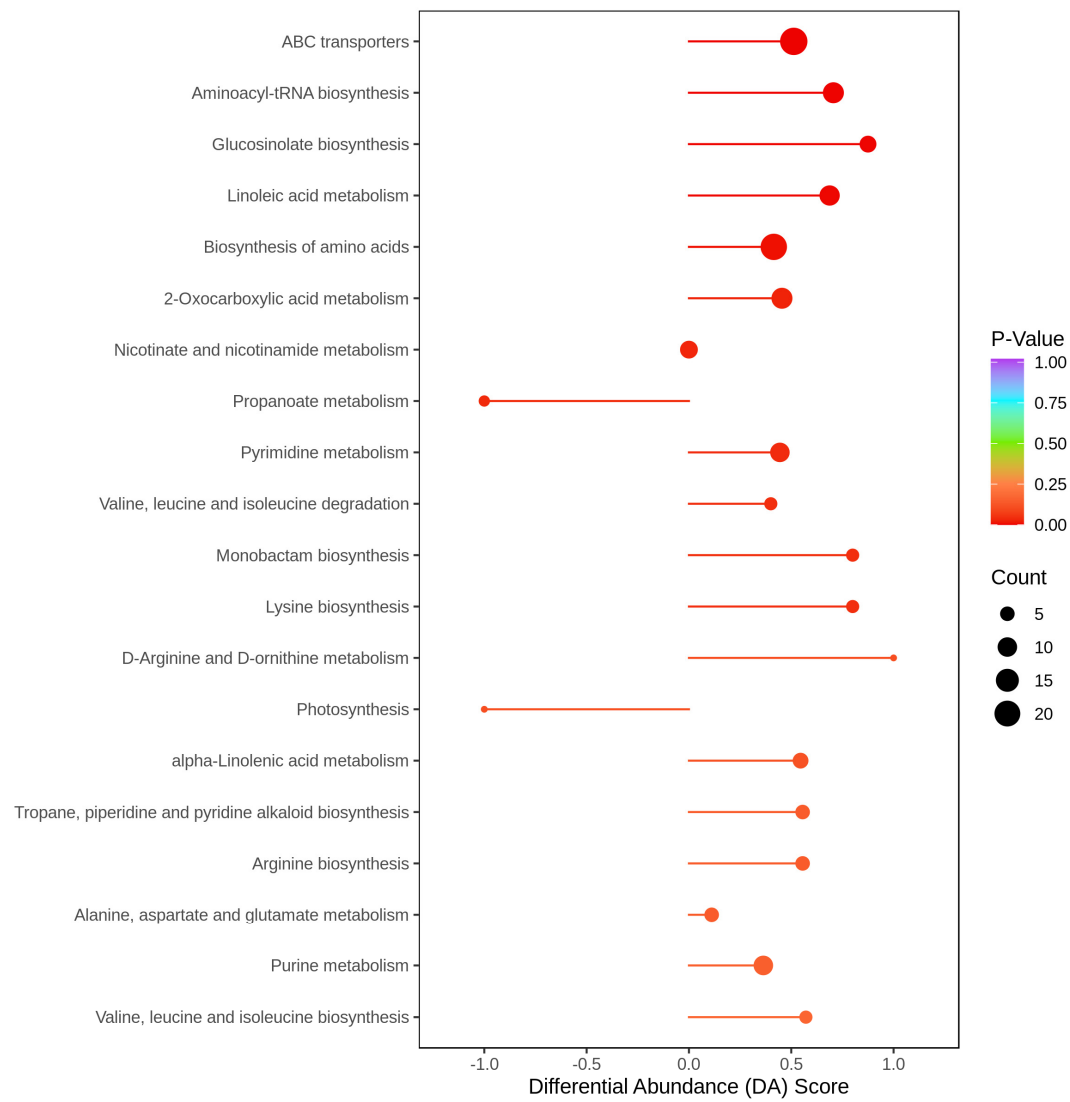

Figure S2: Differential abundance score graph.

Supplement: Supplementary file 1 [file biomolecules-12-00977-s001.zip › Fig.S2.pdf]
